# Analysis and Evaluation of Muscle Quality in Different Parts of the Bighead Carp (*Aristichthys nobilis*)

**DOI:** 10.3390/foods12244430

**Published:** 2023-12-10

**Authors:** Jiaqi Peng, Xiaorong Lu, Ruiqi Fan, Yuanyuan Ren, Huiwu Sun, Yali Yu, Bo Cheng

**Affiliations:** 1 College of Fisheries and Life Science, Shanghai Ocean University, 999 Hucheng Ring Road, Nanhui New Town, Pudong New Area, Shanghai 201306, China; 2 Chinese Academy of Fishery Sciences, No.150, Qingta West Road, Fengtai District, Beijing 100141, China; 3 College of Fisheries, Huazhong Agricultural University, Wuhan 430070, China; 4 Yangtze River Fisheries Research Institute, Chinese Academy of Fishery Sciences, Wuhan 420322, China

**Keywords:** *Aristichthys nobilis*, different parts, nutritional composition, volatile component

## Abstract

In this study, the bighead carp (*Aristichthys nobilis*) was the object of research to compare and analyze the contents of conventional nutrients, amino acids, fatty acids, inosinic acid, and earthy-smelling compounds (geosmin and 2-methylisoborneol) in muscles of its dorsal, belly, tail, opercula, eye socket, and mandible in order to evaluate their quality. The findings could inform recommendations for the consumption and processing of different muscle parts of the bighead carp. The results showed that the water content in the abdominal muscle was significantly lower than that in other parts, and the crude fat content was significantly higher than that in other parts (*p* < 0.05, the same below). Seventeen kinds of amino acids were detected in the muscles of the six parts of the fish, and the dorsal muscles had the highest umami amino acids, essential amino acids and total amino acids, which were 6.45 g/100 g, 6.82 g/100 g and 17.26 g/100 g, respectively. The total amount of essential amino acids in the muscle was higher than that in the FAO/WHO standard model. According to the AAS standard, the first limiting amino acid in the muscle of the six parts was valine (Val). There were 26 kinds of fatty acids in the abdomen, under the gill cover and in the eye socket muscles, and the content of polyunsaturated fatty acids in the mandibular muscles was the highest (45.41%). The content of inosine in the dorsal muscle was significantly higher than that in other parts. Geosmin was the main substance in the muscle. There was no correlation between the distribution of earthy-smelling compounds and fat content, but the content of earthy-smelling compounds in the muscle of the belly and eye socket was the highest. Therefore, the muscle quality of different parts of the bighead carp has its own characteristics, and targeted development and utilization can make more efficient use of bighead carp resources.

## 1. Introduction

*Aristichthys nobilis* is commonly known as chubby fish, bighead fish and baotou fish, as one of “the four major Chinese carps”, and is a unique freshwater economic fish in China. The species is extensively distributed across a range of freshwater habitats nationwide, spanning northern and southern regions. The bighead carp exhibits rapid growth, strong disease resistance, and high nutritional value, making it highly sought after in the market. In 2022, bighead carp production reached 3.269 million metric tons, securing its position as the third-most-produced species, following the grass carp (*Ctenopharyngodon idellus*) and the silver carp (*Hypophthalmichthys molitrix*). This volume far surpassed the production of other common freshwater species, such as the black carp (*Mylopharyngodon piceus*), common carp (*Cyprinus carpio*), and crucian carp (*Carassius auratus*) [1].

The bighead carp is primarily sold fresh, with the edible portion constituting 67.3% of its total weight. It is renowned for its richness in docosahexaenoic acid (DHA), eicosapentaenoic acid (EPA), and essential amino acids [2], which makes it a relatively good source of such nutrients. In China, it serves as a fundamental ingredient in various traditional culinary preparations and enjoys considerable consumer preference [3].

Although the head of the bighead carp is widely favored by consumers, the other parts of its body are typically sold at lower prices. The prevalence of intermuscular bones and pronounced earthy odors contributes to this diminished marketability. This presents significant challenges, including a low processing yield, market volatility, and marginal product value addition, which cumulatively impede the growth of the bighead carp’s aquaculture and processing sectors [4].

The shift in consumer preferences towards convenient, ready-to-cook freshwater fish products, such as fillets, tails, and heads, has been catalyzed by increasing disposable incomes and a faster pace of life [5]. Segmenting products not only facilitates transportation but also allows for the strategic processing of less desirable sections, based on their unique nutritional profiles, to optimize their commercial worth. Regarding the nutritional components of the bighead carp, the head and dorsal of the bighead carp were analyzed. It was found that the content of essential amino acids in the muscle of the bighead carp was above 40%, and that the content of calcium in dorsal meat was the highest, reaching 1195.36 mg/kg [6]. The nutritional composition differences among *Aristichthys nobilis* muscles, bones, skin, scales, viscera, and fin rays have been examined, with fish skin displaying the highest crude protein content of 24.28%, while the viscera and bones showed the lowest crude protein content [7]. The composition of volatile components in Nanwan bighead carp was analyzed, and 41 flavor substances were detected in the muscle, with the highest number being aldehydes (49.15%), which results in the unique flavor of bighead carp [8]. In addition to volatile substances, nucleotides also have a significant impact on the flavor of bighead carp [9]. Inosine monophosphate (IMP), also known as inosinic acid, is a nucleotide that is widely recognized for its role in the flavor of foods, particularly its contribution to the umami taste [10]. However, apart from its unique flavor, bighead carp also has a relatively noticeable earthy and musty odor, which can be attributed to geosmin (GSM) and 2-methylisoborneol (2-MIB) [11]. GSM and 2-MIB, known for imparting earthy-musty scents, are common in freshwater fish, and have a significant impact on the earthy and musty odor [12]. A previous study determined the content of earthy-smelling compounds in the entire bighead carp. It was discovered that the average level of these compounds in bighead carp is 5.4 μg/kg, a concentration significantly exceeding the smell threshold for earthy odors in fish [13].

Although prior research has shed light on the nutritive and volatile components and earthy odor compounds of the bighead carp, comprehensive analysis pertaining to the distribution of these compounds, particularly the geosmin and 2-MIB, across different fish parts is absent. Thus, the present study endeavors to conduct a thorough examination of the nutritional constituents, IMP, GSM, and 2-MIB levels in different sections of six bighead carp, coupled with an assessment of their quality. The objective is to generate pivotal nutritional insights for bighead carp consumption and to provide empirical support for the judicious utilization of its various segments.

## 2. Materials and Methods

### 2.1. Sample Collection and Preparation

**Figure 1:**
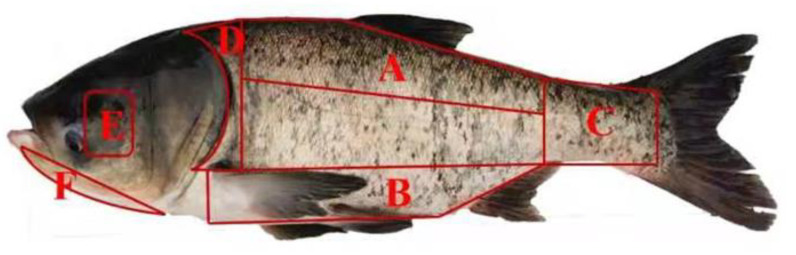
The sampling sites of muscle of a bighead carp. A: dorsal muscle; B: belly muscle; C: tail muscle; D: opercula muscle; E: eye socket muscle; F: mandible muscle.

Six live bighead carp (2.0 ± 0.1 kg) were purchased from Xinzhu Road Vegetable Market in Wuhan City, and they were cultured using semi-intensive aquaculture practices. The muscle tissues from different parts were collected from the dorsal, belly, tail, below the opercula, the eye socket, and the mandible and homogenized (*n* = 6) for each part. The dorsal, belly, and tail muscles were considered parts of the trunk, while the opercula, eye socket, and mandible were categorized as parts of the head. This categorization is used to analyze whether differences still exist between the head and trunk regions for various parameters. The sampling sites are indicated in Figure 1.

### 2.2. Determination of Muscle Composition

The determination of moisture content adhered to the protocol specified in the China National Standard GB/T 5009.3-2016 [14], involving a thermal procedure at 105 °C. For ascertaining ash content, the methodology conformed to the standard GB/T 5009.4-2016 [15]. This entailed the complete carbonization of the sample, followed by transfer to a muffle furnace maintained at 550 °C for high-temperature ashing. Crude protein content was quantified via the Kjeldahl method, a reference method for nitrogen analysis. Finally, the estimation of crude fat content was conducted in accordance with GB 5009.6-2016 [16], utilizing a Soxhlet extraction method with petroleum ether as the solvent.

### 2.3. Amino Acid Content Determination and Quality Evaluation

The determination of amino acid content was based on the reference method GB 5009.124-2016 by the L-8900 automatic amino acid analyzer (Hitachi Ltd., Tokyo, Japan). The nutritional evaluation of amino acids was conducted according to the FAO/WHO (1973) pattern and the amino acid pattern of whole egg protein [17,18]. The amino acid score (AAS), chemical score (CS), and essential amino acid index (EAAI) were calculated using the following formulas [19]:

(1) $$AAS=\frac{Content\;of\;a\;specific\;amino\;acid\;in\;the\;sample\;(mg/gN))}{\left(Content\;of\;the\;corresponding\;amino\;acid\;in\;the\;FAO/WHO\;pattern\;(mg/gN)\right)}$$

(2) $$CS=\frac{Content\;of\;a\;specific\;amino\;acid\;in\;the\;sample\;(mg/gN)}{Content\;of\;the\;same\;amino\;acid\;in\;whole\;egg\;protein\;(mg/gN)}$$

(3) $$EAAI=\sqrt[{n}]{\frac{100A}{AE}\times \frac{100B}{BE}\times \frac{100C}{CE}\times \cdot \cdot \cdot \times \frac{100H}{HE}}$$

Note: n represents the number of essential amino acids being compared, A, B, C, ...; H represent the content of essential amino acids in the sample protein (%); AE, BE, CE, ..., HE represent the content of essential amino acids in whole egg protein (%).

### 2.4. Determination of Fatty Acids

The determination of fatty acid content was based on the reference method GB 5009.168-2016 “National Food Safety Standard—Determination of Fatty Acids in Foods” [20]. The sample preparation process can be briefly summarized as follows: A 2 g sample was weighed and hydrolyzed with 10 mL of 8.3 mol/L hydrochloric acid in a water bath at 70 °C to 80 °C for 40 min. Post-hydrolysis, the sample was extracted with 50 mL of an ether–petroleum ether mixture (1:1, *v*/*v*). The extract was then rotary evaporated to dryness, and the residue was dissolved in 8 mL of 2% sodium hydroxide in methanol solution. Subsequently, 7 mL of 15% boron trifluoride in methanol solution was added, and the mixture was refluxed in a water bath at 80 °C to facilitate the methylation of fatty acids. Finally, the methylated fatty acids were extracted with 5 mL of n-heptane. An amount of 1 mL of the upper layer of the resultant solution was then collected and placed in a sample vial for analysis.

The sample was determined by an Agilent 7890A gas chromatograph equipped with flame ionization detector (FID) (Agilent Technologies, Santa Clara, CA, USA). The instrumental conditions were set as follows: The temperature of the injector was maintained at 270 °C, and the FID temperature was set at 280 °C. The temperature program was established with an initial temperature of 100 °C, held for 13 min; this was followed by a ramp up from 100 °C to 180 °C at a rate of 10 °C/min, with a hold time of 6 min. The temperature was then increased from 180 °C to 200 °C at a rate of 1 °C/min, holding for 20 min, and finally from 200 °C to 230 °C at a rate of 4 °C/min, with a hold time of 10.5 min. The fatty acid content was determined using the external standard method, and the fatty acid methyl ester standards were purchased from Shanghai ANPEL Scientific Instrument Co., Ltd. (Shanghai, China).

### 2.5. Determination of Inosine Monophosphate (IMP)

To determine IMP, a 0.4 g sample was taken and transferred to a 50 mL centrifuge tube. Then, 20 mL of 10% perchloric acid was added, followed by vortex shaking for 1 min. The tube was centrifuged at 8000 rpm for 10 min at 4 °C. The extraction was repeated twice, and the supernatants were combined. The pH was adjusted to 6.0 with 10 mol/L NaOH and then further adjusted to 6.0–6.4 using 1 mol/L NaOH. After pH adjustment, the solution was transferred to a 50 mL volumetric flask and diluted with deionized water at 4 °C. The mixture was centrifuged at 8000 rpm for 10 min at 4 °C, and the supernatant was filtered through a 0.22 μm filter membrane and then analyzed using a Thermo TSQ QUANTUM LC/MS analyzer (Thermo Fisher Scientific, Waltham, MA, USA)

### 2.6. Determination of Geosmin and 2-Methylisoborneol

Samples weighing 0.1 g were put into headspace vials. Then, 1 mL of saturated NaCl solution and 0.15 g NaCl were added, and the bottle was sealed with a C10 rotor. The headspace vial was placed on a magnetic stirrer and incubated at 70 °C for 30 min, and an extraction needle was inserted into the headspace vial. The fiber was then inserted into the injection port of the gas chromatograph-mass spectrometer (GC-MS) for 5 min of sample desorption. 

The gas chromatograph-mass spectrometer was equipped with an HP-5MS capillary column (30 mm × 0.25 mm × 0.25 μm). The injection port temperature was set at 250 °C. The temperature program was as follows: initial temperature of 60 °C, held for 2 min, increased at a rate of 10 °C/min to 120 °C, then increased at a rate of 3 °C/min to 150 °C, held for 1 min, and finally increased at a rate of 20 °C/min to 260 °C, held for 1 min (total time 25.5 min). Helium was used as the carrier gas at a flow rate of 1 mL/min without splitting. The mass spectrometer conditions were as follows: ion source temperature of 230 °C, transfer line temperature of 280 °C. The detection was performed in electron impact (EI) mode using single ion monitoring (SIM), with characteristic m/z values of 112.0 for geosmin and 95.0 for 2-MIB.

### 2.7. Statistical Analysis

Statistical analysis of the data was performed using SPSS 26.0 and was presented as mean ± standard deviation (X ± SD). To verify the normality and homogeneity of variances of the data, the Shapiro–Wilk test and Levene’s test were used. Depending on whether these conditions were met, one-way ANOVA with a post-hoc LSD (least significant difference) test was applied for normally distributed data, or the Kruskal–Wallis non-parametric test followed by a post-hoc Bonferroni test was used otherwise. Different letters indicate significant differences (*p* < 0.05).

## 3. Results

### 3.1. Muscle Composition Variation

The contents of moisture, ash, crude protein, and crude fat in the muscles of different parts of bighead carp are detailed in Table 1. The belly muscle exhibited a significantly lower moisture content compared to the other parts (*p* < 0.05). The lowest values of ash content and crude protein were found in the belly muscle. In contrast, the belly muscle contained a crude fat content of 28.65%, markedly higher than the other parts, with a factor of 24.49 times greater than that of the dorsal muscle, which had a crude protein content of 17.51% and a crude fat content slightly lower than that of the head muscles.

### 3.2. Amino Acids Distribution

Seventeen amino acids were detected in all muscle samples, which are categorized into essential, non-essential, and semi-essential amino acids as shown in Table 2. Dorsal and tail muscles had higher levels of umami taste, essential, and non-essential amino acids than belly muscles. The total umami amino acid content (aspartic acid, glutamic acid, glycine, and alanine) was higher in these parts. The muscles under the opercula had a higher amino acid content compared to the eye socket and mandible muscles but a slightly lower content than in the dorsal and tail muscles.

### 3.3. Essential Amino Acids and Protein Quality

As Table 3 indicates, the total amount of essential amino acids in the muscle tissues of different parts of the bighead carp ranged from 2546 to 3032 mg/g N, and all were higher than the amino acid standards recommended by FAO/WHO (2190 mg/g N). The total essential amino acids in the muscles around the eye socket and gill cover were the closest to the standard of whole egg protein (2960 mg/g N). The amino acid score (AAS), chemical score (CS), and essential amino acid index (EAAI) for the muscles of different parts of the trunk are presented in Table 4. There are certain differences in the limiting amino acids of the muscles from different parts of the trunk under various scoring standards. When evaluated using the AAS, the first limiting amino acid in the muscles of all six regions was valine (Val), with the second limiting amino acid being threonine (Thr) for the eye socket, gill cover, lower jaw, and tail muscles, while for the dorsal and abdominal muscles it was isoleucine (Ile). When utilizing the chemical score (CS) method, the amino acid with the lowest CS is identified as the first limiting amino acid, and the one with the second lowest CS is identified as the second limiting amino acid. In this context, methionine (Met) + cysteine (Cys) were the first limiting amino acids for the dorsal and tail muscles, with Val being the second. For the eye socket, gill cover, lower jaw, and abdominal muscles, Val was the first limiting amino acid. With the exception of the lower jaw muscle, where Met + Cys was the second limiting amino acid, the second limiting amino acid for the remaining three parts was Ile.

### 3.4. Fatty Acid Profile

The fatty acid analysis presented in Table 5 showed 26 types of fatty acids in the belly, under opercula, and eye socket muscles, while the tail muscle had 24, and the dorsal and mandible muscles contained 20 types. As for the content and significant differences between sections of the bighead carp, it is indicated that the belly muscle had high levels of SFAs (saturated fatty acids, 33.12%) and MUFAs (monounsaturated fatty acids, 31.93%), with the PUFAs (polyunsaturated fatty acids) relatively high in the mandibular muscle (45.41%) and dorsal muscle (44.99%). The distribution of fatty acids in different sections of the bighead carp showed variation. SFAs exhibit a consistent presence across both trunk and head regions, but with some variations. In the trunk, the mean SFA content is slightly higher in the belly section (33.12%) and tail sections (32.33%) compared to the dorsal (30.89%). This suggests a marginally richer SFA concentration in the belly area. In the head, there were no significant differences between each section. 

Regarding MUFAs, the highest content of MUFAs was found in the belly muscle (31.93%), with slightly lower levels in the tail, opercula, and eye socket muscles, which were relatively close to each other (27.77%, 29.99%, and 29.96%, respectively). The lowest content was in the mandible (22.69%), followed by the dorsal muscle (24.13%). PUFAs exhibit a trend opposite to that of MUFAs, with relatively higher concentrations in the dorsal (45.41%) and mandible (44.99%) muscles, and the lowest in the belly muscle (34.96%). This trend is consistent across n-3 PUFA, n-6 PUFA, and DHA (docosahexaenoic acid, C22:6n3), with the highest concentrations found in the mandible muscle and the lowest in the belly muscle. As for EPA (eicosapentaenoic acid, C20:5n3), its distribution across various parts showed no significant differences, remaining within the range of 7.55% to 9.52%. 

### 3.5. Flavor Compound Concentrations

As shown in Table 6, the IMP (inosine monophosphate) content in bighead carp muscles was highest in the dorsal muscle at 7.76 mg/g. The mandibular muscle followed with 5.65 mg/g, and the belly muscle had the lowest content at 1.86 mg/g. The distribution of IMP in the head and trunk regions does not show a specific trend.

### 3.6. Earthy Odor Determinants

GSM and 2-MIB were detected in all six muscle parts (Table 7). The concentrations of these compounds were notably higher in the belly and eye socket muscles, with 2-MIB at 0.19 μg/kg and GSM at 1.44 μg/kg in the belly muscles and 1.85 μg/kg in the eye socket muscles. These levels exceed the sensory thresholds of 0.085 μg/kg for 2-MIB and 0.59 μg/kg for GSM in fish [21]. In the tail and mandible muscles, the levels of GSM (0.28 μg/kg in the tail, 0.35 μg/kg in the mandible) and 2-MIB (both 0.07 μg/kg) were relatively low, with neither exceeding the sensory thresholds. 

## 4. Discussion

### 4.1. Muscle Composition Variation

The findings regarding the muscle composition of the bighead carp have several implications. The high crude fat content of the belly muscle, coupled with its simple component profile, few impurities, and richness in polyunsaturated fatty acids [22], underscores its potential as a raw material for fish oil processing. This could significantly contribute to the advancement of the deep processing industry of freshwater fish, thereby enhancing economic benefits. Moreover, given that the processing of freshwater fish predominantly targets surimi and its products, the utilization of by-products such as the belly, head, and viscera—which constitute 40–55% [23] of the raw fish—is of great interest.

In contrast, the crude protein content in other parts of the bighead carp, ranging from 12.30% to 17.51%, and the crude lipid content between 1.17% and 4.05%, categorizes these areas as low-fat (oil content <5%) according to Stansby [24]. This distinction is vital for nutritional exploitation, as the dorsal (17.51%) and tail (16.60%) sections, with their high protein content, are ideal for protein or amino acid extraction. The moisture content across different parts of the carp varies from 60.81% to 83.09%, and the ash content from 0.80% to 1.34%. These moisture and ash levels are consistent with findings for other species, such as the common carp (77.20% moisture, 1.30% ash) reported by Mohanty et al. [25] and the African catfish (75.92% moisture, 0.83% ash) by Teame et al. [26]. For mineral extraction, the tail and dorsal sections, with the highest ash content, emerge as promising areas.

However, the reasons for the higher fat content in the belly of the bighead carp and strategies for its regulation require further research, especially due to limited studies segmenting fish into such detailed parts for nutritional comparison. Overall, the data present a clear stratification of nutritional components across different sections of the bighead carp, offering valuable insights for targeted nutritional exploitation.

### 4.2. Amino Acids Distribution

The nutritional quality of the muscle proteins is gauged by the type, content, and ratio of amino acids present. The delicacy of muscle is largely influenced by umami amino acids, such as aspartic acid, glutamic acid, glycine, and alanine, with the content of glutamic acid being particularly pivotal for the umami taste [27]. Analysis of the amino acids in bighead carp muscles revealed that the dorsal and tail muscles not only had a higher glutamic acid content (2.69 g/100 g and 2.50 g/100 g, respectively) but also exhibited significantly greater total, essential, and umami amino acid levels compared to other parts. This suggests that the dorsal and tail portions of the bighead carp could offer superior taste and nutritional benefits.

### 4.3. Essential Amino Acids and Protein Quality

The protein quality of bighead carp muscles is considerably influenced by their anatomical location. When compared with different types of bighead carp, the EAAI for trunk muscles in this study was found to be superior to that of red and black bighead carp (80.47 and 80.13) as reported by Liu et al. [28], but inferior to that for bighead carp from the Miyun Reservoir (98.76) as documented by Jia et al. [29]. These muscles were, however, on par with American bighead carp (92.69) as per the findings of Ma et al. [30]. Age-related variations in EAAI were also evident in the study by Zheng et al. [31]. Furthermore, the disparity in limiting amino acids in the muscles, especially Val, Thr, Ile, and Met+Cys, as opposed to other regions and studies, suggests that the nutritional content is not only species-specific but also significantly affected by environmental conditions and cultivation practices, as seen in comparisons with bighead carp from the Miyun Reservoir (Trp, Val) [29], Shanxi Reservoir (Trp, Val, Met, and Cys) [31], and in cultivated bighead carp (Val, Met, and Cys) [32]. Additionally, the limiting amino acids (Val, Thr) in the three head muscle parts studied here show differences compared to the limiting amino acids (Met, Cys, and Val) in the head of bighead carp as measured by Jiang et al. [6], which indicates that the head muscle limiting amino acids vary. This suggests that further research is needed to compare the effects of various factors such as temperature, water quality, and nutritional intake on the nutritional composition of bighead carp muscle.

### 4.4. Fatty Acid Profile

The muscle tissue of bighead carp presents a beneficial fatty acid composition, with an adequate balance of saturated (SFAs), monounsaturated (MUFAs), and polyunsaturated fatty acids (PUFAs). With unsaturated fatty acids accounting for 66.88–69.11% of the total fatty acids across the six muscle parts analyzed, the muscles of the mandible and dorsal part exhibit the highest content of these beneficial fats. This finding indicates that the nutritional quality of the dorsal muscle is comparable, if not superior, to that of the head muscle, which has been traditionally preferred by consumers for its rich protein and unsaturated fat content [33]. The mandibular muscle, with the highest percentage of EPA and DHA at 25.63%, surpasses the content found in other fish species like the grass carp (9.43%), carp (13.91%), and tilapia (*Oreochromis mossambicus*) (11.49%) [34]. The significant presence of PUFAs in the mandibular and dorsal muscles, representing 45.41% and 44.99%, respectively, suggests that these parts have high fatty acid nutritional value and contribute to the flavor profile of the fish, which correlates with findings on flavor enhancement by PUFA in muscle [35].

### 4.5. IMP Content and Flavor Enhancement

The importance of IMP as a key umami substance has been well documented, especially its role in enhancing the flavor of fish when combined with adenosine diphosphate [36,37]. In the present study, IMP was highest in the dorsal muscle, followed by the mandibular muscle, and the belly muscle had the lowest concentration. These findings are consistent with the IMP contents in different fish species and tissues, as reported in previous studies by Chen et al. [38]. Except for belly muscles, all other examined muscle parts of bighead carp had an IMP content exceeding 3.24 mg/g. This is notably higher than in other fish species, such as Siniperca chuatsi (*Hypophthalmichthys molitrix*) with 2.46 mg/g [39], the grass carp with 2.18 mg/g [40], and the silver carp with 0.91 mg/g [41], highlighting the superior flavor profile of bighead carp. These findings indicate that not only does the IMP content vary significantly between different species and within the tissues of the same species, but also that the bighead carp, particularly its dorsal and mandibular muscles, can be a superior choice for consumers seeking the umami taste in their seafood.

### 4.6. Earthy Odor Compounds

GSM and 2-MIB were notably higher in the belly and eye socket muscles in our research. These levels exceed the sensory thresholds of 0.085 μg/kg for 2-MIB and 0.59 μg/kg for GSM in fish [21], emphasizing the significant presence of GSM, which ranges from 0.15 to 1.85 μg/kg in bighead carp muscles, contributing to the earthy odor. However, the levels of GSM and 2-MIB are relatively low, with neither exceeding the sensory thresholds. This suggests that these two parts may have a relatively lighter earthy and musty odor. Comparative analysis of GSM and 2-MIB content with other studies indicates that bighead carp might have higher concentrations of these earthy-smelling compounds than other fish species in different conditions reported in literature. For instance, Atlantic salmon in recirculating aquaculture systems (RAS) showed 2-MIB concentrations of 0.55 to 0.99 μg/kg and GSM concentrations of 0.26 to 0.51 μg/kg [42]. Furthermore, tilapia cultured in Hainan ponds displayed GSM levels of 0.11 to 0.28 μg/kg and 2-MIB levels of 0.08 to 0.72 μg/kg [43], which are lower than those found in bighead carp. The findings from this study underscore a pronounced earthy odor issue in bighead carp that could affect its consumer acceptability.

The effect of fish fat content on the concentration of these compounds has been explored in other research [21,44]. While some studies, such as that by Tucker [45], have found a correlation between higher fat content and stronger earthy odors, this study did not find a significant correlation between the crude fat content in various parts of bighead carp muscle and the concentration of earthy-smelling compounds (*p* > 0.05). This lack of correlation echoes the conclusions of Petersen et al. [46], who found no correlation between fat content and the concentration of off-flavor compounds. Such findings suggest that the mechanisms governing the accumulation of GSM and 2-MIB in bighead carp may be different from those in other species or may be influenced by factors other than fat content. Given the economic importance of bighead carp, understanding and mitigating the factors contributing to off-flavors is crucial for improving its marketability and ensuring its palatability for consumers.

## 5. Conclusions

The dorsal and tail muscles of bighead carp showcase a desirable profile of high protein (17.51% and 16.60%, respectively) and low fat (1.17% and 3.07%, respectively). Essential amino acids in these muscles meet and surpass FAO/WHO standards. Unsaturated fatty acids are prevalent, accounting for 66.88–69.11% of total fatty acids, with the highest concentrations found in mandible and dorsal muscles, affirming their nutritional worth. The dorsal muscle stands out for its high IMP content (7.76 mg/g), contributing to flavor. While the belly and eye socket muscles exhibit elevated levels of GSM and 2-MIB, compounds responsible for the fish’s earthy scent, this issue still requires further investigation in subsequent research. Different parts of bighead carp muscle possess distinct qualities, suggesting specialized approaches to their use could enhance resource utilization. Future research could explore targeted applications, such as harnessing protein-rich areas for nutritional enhancements and tapping into lipid-dense sections for oil extraction. Advancements in aquaculture practices to optimize the nutritional quality of bighead carp will be a key area of focus. These approaches hold the potential to maximize the value derived from this species, aligning with sustainable and efficient resource utilization.

## Figures and Tables

**Table 1:** Essential nutrient components of muscle in different parts of *Aristichthys nobilis* (% wet weight).

Index	Trunk			Head		
	Dorsal	Belly	Tail	Opercula	Eye Socket	Mandible
Moisture	79.51 ± 0.71 ^a^	60.81 ± 5.13 ^b^	76.51 ± 4.75 ^a^	78.96±0.43 ^a^	80.98 ± 3.32 ^a^	83.09 ± 0.41 ^a^
Ash	1.26 ± 0.02 ^ab^	0.80 ± 0.01 ^c^	1.34 ± 0.25 ^a^	1.03±0.13 ^bc^	1.19 ± 0.03 ^ab^	1.05 ± 0.06 ^bc^
Crude protein	17.51 ± 0.46 ^a^	12.30 ± 0.22 ^d^	16.60 ± 0.65 ^ab^	15.77±0.52 ^bc^	12.36 ± 1.48 ^d^	14.76 ± 0.18 ^c^
Crude lipid	1.17 ± 0.14 ^c^	28.65 ± 0.08 ^a^	3.07 ± 1.20 ^b^	3.47±0.65 ^b^	4.05 ± 0.98 ^b^	3.66 ± 0.07 ^b^

Note: distinct letters denote statistically significant differences (*p* < 0.05).

**Table 2:** The amino acid content in muscle of different parts of *Aristichthys nobilis* (g/100 g wet weight).

Amino Acid	Trunk			Head		
	Dorsal	Belly	Tail	Opercula	Eye Socket	Mandible
Aspartic acid Asp ^△^	1.82 ± 0.11 ^a^	1.04 ± 0.15 ^d^	1.65 ± 0.01 ^ab^	1.37 ± 0.06 ^bc^	1.13 ± 0.20 ^cd^	1.34 ± 0.03 ^c^
Glutamic acid Glu ^△^	2.69 ± 0.13 ^a^	1.54 ± 0.18 ^e^	2.50 ± 0.00 ^ab^	2.20 ± 0.08 ^bc^	1.77 ± 0.28 ^de^	2.08 ± 0.01 ^cd^
Glycine Gly ^△^	0.96 ± 0.04 ^b^	0.68 ± 0.00 ^c^	1.15 ± 0.01 ^a^	0.87 ± 0.06 ^b^	0.95 ± 0.09 ^b^	0.96 ± 0.11 ^b^
Alanine Ala ^△^	0.98 ± 0.06 ^a^	0.60 ± 0.03 ^d^	1.00 ± 0.01 ^a^	0.88 ± 0.01 ^b^	0.78 ± 0.04 ^c^	0.82 ± 0.05 ^bc^
Threonine Thr *	0.80 ± 0.04 ^a^	0.51 ± 0.01 ^d^	0.76 ± 0.00 ^ab^	0.74 ± 0.01 ^b^	0.57 ± 0.02 ^c^	0.61 ± 0.00 ^c^
Valine Val *	0.83 ± 0.05 ^a^	0.52 ± 0.08 ^c^	0.84 ± 0.03 ^a^	0.70 ± 0.03 ^ab^	0.60 ± 0.12 ^bc^	0.68 ± 0.04 ^abc^
Methionine Met *	0.29 ± 0.01 ^b^	0.08 ± 0.04 ^d^	0.28 ± 0.04 ^b^	0.40 ± 0.00 ^a^	0.19 ± 0.03 ^c^	0.32 ± 0.01 ^b^
Isoleucine Ile *	0.78 ± 0.04 ^a^	0.45 ± 0.04 ^c^	0.79 ± 0.01 ^a^	0.81 ± 0.00 ^a^	0.61 ± 0.04 ^b^	0.63 ± 0.06 ^b^
Leucine Leu *	1.47 ± 0.08 ^a^	0.91 ± 0.06 ^c^	1.40 ± 0.02 ^a^	1.38 ± 0.04 ^a^	1.07 ± 0.10 ^b^	1.13 ± 0.04 ^b^
Phenylalanine Phe *	0.84 ± 0.05 ^a^	0.70 ± 0.06 ^bc^	0.80 ± 0.02 ^ab^	0.82 ± 0.04 ^a^	0.63 ± 0.08 ^c^	0.61 ± 0.01 ^c^
Lysine Lys *	1.65 ± 0.08 ^a^	0.92 ± 0.13 ^d^	1.52 ± 0.04 ^ab^	1.23 ± 0.04 ^bc^	1.03 ± 0.23 ^cd^	1.25 ± 0.06 ^bc^
Histidine His #	0.54 ± 0.04 ^a^	0.27 ± 0.01 ^e^	0.47 ± 0.00 ^ab^	0.43 ± 0.01 ^bc^	0.32 ± 0.05 ^de^	0.37 ± 0.01 ^cd^
Arginine Arg #	1.04 ± 0.04 ^a^	0.71 ± 0.02 ^e^	1.05 ± 0.01 ^a^	0.97 ± 0.01 ^b^	0.84 ± 0.01 ^d^	0.92 ± 0.03 ^b^
Cysteine Cys	0.45 ± 0.02 ^b^	0.45 ± 0.11 ^b^	0.48 ± 0.02 ^b^	0.92 ± 0.09 ^a^	0.58 ± 0.27 ^ab^	0.37 ± 0.07 ^b^
Serine Ser	0.76 ± 0.04 ^a^	0.47 ± 0.04 ^d^	0.73 ± 0.01 ^ab^	0.63 ± 0.01 ^bc^	0.56 ± 0.08 ^cd^	0.60 ± 0.01 ^c^
Tyrosine Tyr	0.70 ± 0.04 ^a^	0.47 ± 0.09 ^c^	0.67 ± 0.01 ^a^	0.65 ± 0.02 ^ab^	0.50 ± 0.07 ^c^	0.54 ± 0.02 ^bc^
Proline Pro	0.65 ± 0.03 ^b^	0.59 ± 0.07 ^b^	0.76 ± 0.04 ^b^	1.00 ± 0.06 ^a^	0.79 ± 0.15 ^b^	0.64 ± 0.06 ^b^
UAA	6.45 ± 0.31 ^a^	3.87 ± 0.36 ^d^	6.29 ± 0.02 ^a^	5.31 ± 0.07 ^b^	4.61 ± 0.41 ^c^	5.21 ± 0.11 ^bc^
EAA	6.82 ± 0.37 ^a^	4.17 ± 0.21 ^c^	6.55 ± 0.06 ^a^	6.26 ± 0.08 ^a^	4.87 ± 0.52 ^bc^	5.37 ± 0.11 ^b^
NEAA	9.01 ± 0.44 ^a^	5.86 ± 0.30 ^c^	8.94 ± 0.09 ^a^	8.51 ± 0.42 ^a^	7.04 ± 0.16 ^b^	7.36 ± 0.26 ^b^
TAA	17.26 ± 0.88 ^a^	10.94 ± 0.60 ^c^	16.85 ± 0.18 ^a^	15.99 ± 0.06 ^a^	12.91 ± 0.81 ^b^	13.86 ± 0.38 ^b^
EAA/TAA(%)	39.51%	45.62%	38.87%	39.15%	37.72%	38.74%
UAA/TAA(%)	37.37%	35.37%	37.33%	33.21%	35.71%	37.60%

Note: * indicates essential amino acids; △ denotes umami taste amino acid; # stands for semi-essential amino acid. UAA: umami taste amino acids. EAA: essential amino acids. NEAA: non-essential amino acids. TAA: total amino acids. Distinct letters denote statistically significant differences (*p* < 0.05).

**Table 3:** Comparison of muscle essential amino acids of *Aristichthys nobilis* in different parts (mg/g N).

Essential Amino Acid	Trunk			Head			FAO/WHO	Egg Protein
	Dorsal	Belly	Tail	Opercula	Eye Socket	Mandible		
Thr	286	259	286	293	288	258	250	292
Val	296	264	316	277	303	288	310	411
Ile	278	229	297	321	308	267	250	331
Leu	525	462	527	547	541	478	440	534
Lys	589	467	572	487	521	529	340	441
Met + Cys	264	269	286	523	389	292	220	386
Phe + Try	550	595	553	583	571	487	380	565
Total volume	2788	2546	2839	3032	2923	2600	2190	2960

**Table 4:** Comparison of AAS and CS in different parts of *Aristichthys nobilis*.

	Trunk						Head					
	Back		Belly		Tail		Opercula		Eye Socket		Mandible	
	AAS	CS	AAS	CS	AAS	CS	AAS	CS	AAS	CS	AAS	CS
Thr	1.14	0.98	1.04	0.89	1.14 ^▲▲^	0.98	1.17 ^▲▲^	1.00	1.15 ^▲▲^	0.99	1.03 ^▲▲^	0.88
Val	0.96 ^▲^	0.72 ^▲▲^	0.85 ^▲^	0.64 ^▲^	1.02 ^▲^	0.77 ^▲▲^	0.89 ^▲^	0.68 ^▲^	0.98 ^▲^	0.74 ^▲^	0.93 ^▲^	0.70 ^▲^
Ile	1.11 ^▲▲^	0.84	0.91 ^▲▲^	0.69 ^▲▲^	1.19	0.90	1.28	0.97 ^▲▲^	1.23	0.93 ^▲▲^	1.07	0.81
Leu	1.19	0.98	1.05	0.87	1.20	0.99	1.24	1.02	1.23	1.01	1.09	0.90
Lys	1.73	1.34	1.37	1.06	1.68	1.30	1.43	1.11	1.53	1.18	1.56	1.20
Met + Cys	1.20	0.68 ^▲^	1.22	0.70	1.30	0.74 ^▲^	2.38	1.36	1.77	1.01	1.33	0.76 ^▲▲^
Phe + Try	1.45	0.97	1.56	1.05	1.46	0.98	1.53	1.03	1.50	1.01	1.28	0.86
EAAI	91.03		82.75		93.62		100.57		97.36		86.09	

Note: ^▲^ denotes the first limiting amino acid; ^▲▲^ represents the second limiting amino acid.

**Table 5:** Fatty acid composition and content in different parts of *Aristichthys nobilis* (%).

Fatty Acid	Trunk			Head		
	Dorsal	Belly	Tail	Opercula	Eye Socket	Mandible
**C11:0**	-	0.09 ± 0.03 ^a^	0.04 ± 0.06 ^ab^	0.07 ± 0.01 ^ab^	0.07 ± 0.02 ^ab^	-
**C12:0**	0.24 ± 0.01 ^c^	0.43 ± 0.04 ^a^	0.29 ± 0.00 ^bc^	0.35 ± 0.03 ^b^	0.33 ± 0.03 ^b^	0.17 ± 0.03 ^d^
**C13:0**	0.17 ± 0.01 ^b^	0.30 ± 0.05 ^a^	0.22 ± 0.03 ^ab^	0.24 ± 0.04 ^ab^	0.26 ± 0.04 ^ab^	0.17 ± 0.04 ^b^
**C14:0**	4.12 ± 0.13 ^cd^	6.61 ± 0.50 ^a^	4.95 ± 0.21 ^bc^	5.83 ± 0.54 ^ab^	5.57 ± 0.33 ^ab^	3.84 ± 0.54 ^d^
**C15:0**	1.21 ± 0.05 ^bc^	1.66 ± 0.16 ^a^	1.41 ± 0.01 ^abc^	1.48 ± 0.11 ^ab^	1.54 ± 0.14 ^a^	1.19 ± 0.09 ^c^
C16:0	17.84 ± 0.35	18.29 ± 0.19	18.19 ± 0.68	17.93 ± 0.41	17.80 ± 0.40	18.14 ± 0.18
C17:0	0.98 ± 0.05	1.07 ± 0.17	1.10 ± 0.04	0.99 ± 0.08	1.17 ± 0.03	1.06 ± 0.04
**C18:0**	6.33 ± 0.18^b^	3.96 ± 0.16 ^e^	5.75 ± 0.18 ^c^	4.81 ± 0.20 ^d^	4.80 ± 0.25 ^d^	7.34 ± 0.23 ^a^
C20:0	-	0.36 ± 0.01	0.36 ± 0.01	0.33 ± 0.02	0.33 ± 0.02	-
C22:0	-	0.38 ± 0.01	-	0.38 ± 0.07	0.37 ± 0.07	-
**∑SFAs**	30.89 ± 0.07 ^c^	33.12 ± 0.59 ^a^	32.33 ± 0.58 ^ab^	32.43 ± 0.04 ^ab^	32.25 ± 0.22 ^ab^	31.91 ± 0.24 ^b^
C14:1n5	0.15 ± 0.01	0.28 ± 0.02	0.18 ± 0.05	0.23 ± 0.03	0.25 ± 0.01	0.29 ± 0.17
**C16:1n7**	7.29 ± 0.16 ^c^	10.12 ± 0.00 ^a^	8.64 ± 0.59 ^b^	9.05 ± 0.20 ^b^	9.13 ± 0.01^b^	6.91 ± 0.37 ^c^
C17:1n7	-	-	-	1.00 ± 0.01	1.06 ± 0.01	-
**C18:1n9t**	0.39 ± 0.03 ^b^	0.52 ± 0.06 ^a^	0.45 ± 0.06 ^ab^	0.47 ± 0.05 ^ab^	0.48 ± 0.04 ^ab^	0.40 ± 0.02 ^ab^
C18:1n9c	15.20 ± 2.35	19.18 ± 3.16	17.23 ± 1.77	17.71 ± 2.88	17.62 ± 1.97	13.54 ± 1.44
C22:1n9	-	0.20 ± 0.04	-	-	-	-
C24:1n9	-	-	-	-	-	0.57 ± 0.04
**C20:1**	1.10 ± 0.31 ^ab^	1.65 ± 0.27 ^a^	1.25 ± 0.22 ^ab^	1.53 ± 0.27 ^ab^	1.43 ± 0.23 ^ab^	0.98 ± 0.12 ^b^
**∑ MUFAs**	24.13 ± 2.85 ^bc^	31.93 ± 3.51 ^a^	27.77 ± 2.69 ^abc^	29.99 ± 3.00 ^ab^	29.96 ± 2.20 ^ab^	22.69 ± 1.42 ^c^
**C18:2n6c**	4.78 ± 0.12 ^ab^	5.42 ± 0.40 ^a^	4.90 ± 0.15 ^ab^	5.01 ± 0.31 ^a^	5.01 ± 0.29 ^a^	4.20 ± 0.26 ^b^
C18:3n6	-	0.45 ± 0.01a	0.32 ± 0.01	0.36 ± 0.04	0.37 ± 0.03	-
**C18:3n3**	6.83 ± 0.21 ^cd^	9.36 ± 0.54 ^a^	7.56 ± 0.11 ^bc^	8.54 ± 0.77 ^ab^	8.33 ± 0.51 ^ab^	6.02 ± 0.62 ^d^
C20:2	0.65 ± 0.06	0.64 ± 0.06	0.75 ± 0.10	0.64 ± 0.04	0.69 ± 0.03	0.72 ± 0.05
C20:3n6	-	0.72 ± 0.03	0.72 ± 0.04	0.71 ± 0.01	0.71 ± 0.01	-
C20:3n3	1.18 ± 0.45	0.96 ± 0.13	0.99 ± 0.09	0.99 ± 0.11	1.02 ± 0.09	1.08 ± 0.12
**C20:4n6**	6.51 ± 0.27 ^b^	3.29 ± 0.21 ^e^	5.40 ± 0.63 ^c^	4.22 ± 0.11 ^d^	4.41 ± 0.06 ^d^	7.75 ± 0.48 ^a^
C22:2n6	0.35 ± 0.09	-	-	-	-	-
C20:5n3	9.52 ± 0.64	7.55 ± 0.86	8.07 ± 0.77	7.98 ± 1.02	7.88 ± 0.78	9.43 ± 0.50
**C22:6n3**	15.18 ± 0.94 ^a^	6.55 ± 0.69 ^c^	11.17 ± 1.58 ^b^	9.10 ± 0.64 ^b^	9.36 ± 0.61 ^b^	16.20 ± 0.11 ^a^
**∑PUFAs**	44.99 ± 2.78 ^a^	34.96 ± 2.92 ^b^	39.90 ± 3.26 ^ab^	37.57 ± 3.04 ^b^	37.79 ± 2.43 ^b^	45.41 ± 1.18 ^a^
**∑n-3 PUFA**	32.70 ± 2.23 ^a^	24.42 ± 2.22 ^b^	27.79 ± 2.35 ^ab^	26.62 ± 2.53 ^b^	26.60 ± 2.00 ^b^	32.74 ± 1.34 ^a^
**∑n-6 PUFA**	11.64 ± 0.49 ^ab^	9.89 ± 0.65 ^c^	11.36 ± 0.81 ^ab^	10.32 ± 0.47 ^bc^	10.51 ± 0.40 ^bc^	11.95 ± 0.21 ^a^

Note: - indicates that the data were below the limit of quantification. Distinct letters denote statistically significant differences (*p* < 0.05).

**Table 6:** IMP content in different parts of *Aristichthys nobilis* (mg/g wet weight).

Index	Trunk			Head		
	Dorsal	Belly	Tail	Opercula	Eye Socket	Mandible
IMP	7.76 ± 0.14 ^a^	1.86 ± 0.21 ^de^	3.24 ± 1.54 ^cd^	3.42 ± 0.17 ^cd^	3.35 ± 0.17 ^cd^	5.65 ± 0.99 ^b^

Note: distinct letters denote statistically significant differences (*p* < 0.05).

**Table 7:** GSM and 2-MIB content in different parts of *Aristichthys nobilis* (μg/kg wet weight).

Index	Trunk			Head		
	Dorsal	Belly	Tail	Opercula	Eye Socket	Mandible
2-MIB	0.10 ± 0.01 ^bc^	0.19 ± 0.01 ^a^	0.07 ± 0.00 ^c^	0.16 ± 0.05 ^ab^	0.19 ± 0.02 ^a^	0.07 ± 0.02 ^c^
GSM	0.15 ± 0.02 ^c^	1.44 ± 0.26 ^a^	0.28 ± 0.00 ^c^	0.88 ± 0.20 ^b^	1.85 ± 0.40 ^a^	0.35 ± 0.14 ^bc^

Note: distinct letters denote statistically significant differences (*p* < 0.05).

## Data Availability

Data is contained within the article.

## Abbreviations

AAS	Amino acid score
CS	Chemical score
GSM	Geosmin
2-MIB	2-methylisoborneol
IMP	Inosine monophosphate
